# A Mechanism Study on the (+)-ESI-TOF/HRMS Fragmentation of Some PPI Prazoles and Their Related Substances

**DOI:** 10.3390/molecules28155852

**Published:** 2023-08-03

**Authors:** Luhong Wang, Lixue Chen, Yichen Yao, Hongyan Shen, Youjun Xu

**Affiliations:** Key Laboratory of Structure-Based Drug Design and Discovery (Ministry of Education), School of Pharmaceutical Engineering, Shenyang Pharmaceutical University, Shenyang 110016, China; luhongwang92@163.com (L.W.); chenlx2016@126.com (L.C.); yaoyichen20200729@outlook.com (Y.Y.)

**Keywords:** prazole, related substance, ESI-TOF/HRMS, fragmentation ionization

## Abstract

Fragmentation mechanisms of some prazoles and their related substances were newly investigated in this paper via positive mode ESI-TOF HRMS^1^ and HRMS^2^. Some novel fragmentation rules or ions were found or detected in the research. The pyridine and the benzoimidazole ring remained in most cases during the ionization, and heterolytic fragmentations often occurred near the -S(O)_n_CH_2_- linker to give the [1,3]-H migration ion or [1,7]-H migration ion rearranging across the benzoimdazole ring. Smiles rearrangement ionizations also frequently occurred, initiated by the attack of the lone pair electrons from the pyridine ring, and the sulfones gave special *N*-(2-benzoimdazolyl) pyridine ions (**11b** and **12c**) by a direct extraction from SO_2_, and the thioethers gave similar framework ions (**8c**, **9c** and **10c**) via the rearrangement and a further homolytic cleavage of SH radicals. However, the sulfoxides were seldom detected in the corresponding Smiles rearrangement ions during our measurement, and the *N*′-oxides of the pyridines did not undergo the Smiles rearrangement ionization due to the absence of the lone pair electrons. The 5/6-membered chelating ions with Na^+^ or K^+^ were frequently detected as the molecular and further fragment ions. Some novel and interesting fragment ions containing bivalent (**8b** and **9b**), tetravalent (**4b**, **5c** and **6c**) or hexavalent (**15b** and **16b**) sulfurs were first reported here.

## 1. Introduction

**Figure 1:**
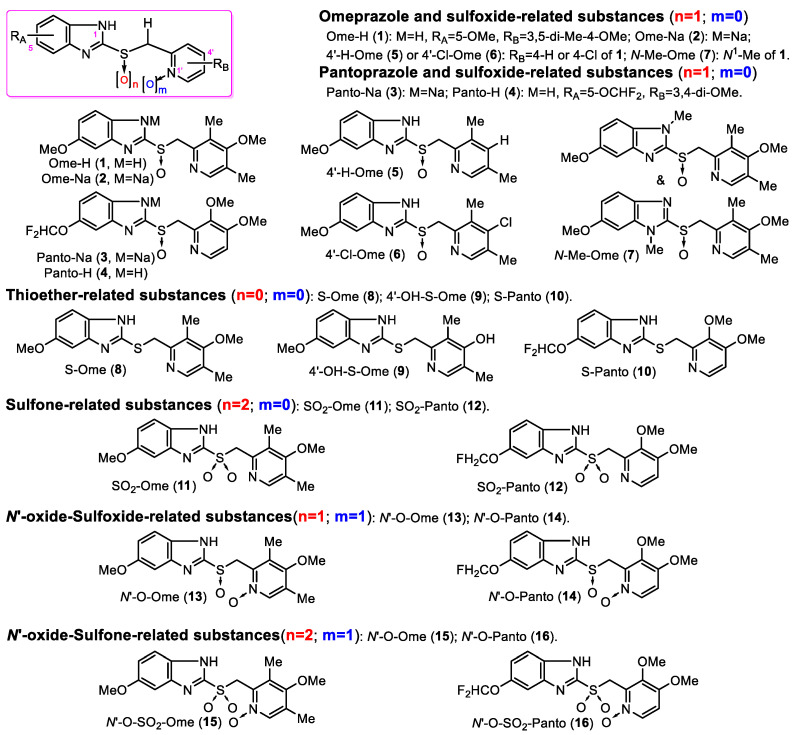
The structures and code names of prazoles & their related substances.

Proton pump inhibitors (PPIs) are now considered the drugs of choice in managing patients with peptic ulcer disease (PUD), gastroesophageal reflux disease (GERD), and Zollinger–Ellison syndrome (ZES) [1,2]. PPIs are the first choice as antiulcer agents in clinical applications. Most oral PPI preparations are enteric-coated to prevent rapid degradation of the drugs in the acidic conditions of the stomach [3,4,5]. PPIs are easy to concentrate in the acidic environment because of the liposoluble and weakly basic properties, and they specifically distribute in the parietal cells [6,7]. They are transformed to active sulfenamide or the corresponding unstable sulfenic acid via Smile rearrangement reactions in the acidic conditions and the sulfur atom can covalently bind with cysteine residues in the position of enzyme H^+^/K^+^-ATPase (hydrogen-potassium adenosine triphosphates) [8,9,10]. The irreversible combination forms an enzyme–inhibitor complex to inactivate the enzyme, and then inhibit the secretion of gastric juice [11]. The PPIs undergo extensive hepatic metabolism and the metabolites are eliminated in the urine and feces [12,13]. PPIs partially undergo metabolism by cytochrome (CYP)2C19, a member of CYP450 known to exhibit genetic polymorphism [14,15]. A small proportion of the Caucasian (2–6%) [16] and Asian (Japanese, 19–23%) [17] populations are poor metabolizers of PPIs due to the genetic polymorphism, which could have some clinical implications. The prazoles have a common structure, 2-((pyridine-2-ylmethyl) sulfinyl)-1*H*-benzo[d]imidazole [18]. We studied five types of prazoles and their related substances (Figure 1), including: the sulfoxides (n = 1 and m = 0, comp. **1**–**7**), the thioethers (n = 0 and m = 0, comp. **8**–**10**), the sulfones (n = 2 and m = 0, comp. **11**–**12**), the sulfinyl nitroxides (n = 1 and m = 1, comp. **13**–**14**), and the sulfonyl nitroxides (n = 2 and m = 1, comp. **15**–**16**). All the related compounds were studied often in the literature and strictly limited in the typical pharmacopoeias.

Very common ionization such as the [M + H]^+^/[M + Na]^+^/[M + K]^+^ ion or [M − H]^–^/[M − Na]^–^ ion was detected for the prazoles, both in positive and negative HRMS^1^ mode, and some fragment ions were reported in the literature [3,18,19]. However, the literature was not comprehensive and systematic, and the definite mechanism of the degree of ions formed was not explained clearly. Thus, it is emphasized and newly reported that some novel special fragmentation patterns or new ion structures have been found, and a reasonable explanation for the detected ions is given via organic chemistry and mass spectrometry theories during our study herein.

## 2. Results and Discussion

Benzimidazole moiety (Ar) on the left and prydine moiety (Py) on the right in the structure of Ar-S(O)_n_CH_2_-Py were relatively stable and the structural characteristics remained in most cases during the ionization, and homolytic and heterolytic fragmentations often occurred near the -S(O)_n_CH_2_- linker to give the [1,3]-H migration ion or [1,7]-H migration ion rearranging across the benzoimdazole ring. Smiles rearrangement ionizations also frequently occurred, initiated by the attack of the lone pair electrons from the pyridine ring. The positive ionization of ESI-TOF HRMS^1^ and HRMS^2^ is mainly emphasized or analyzed in this paper.

### 2.1. Omeprazole (Ome-H) and the Related Substances

#### 2.1.1. Omeprazole (Ome-H) and Omeprazole Sodium (Ome-Na)

**Scheme 1:**
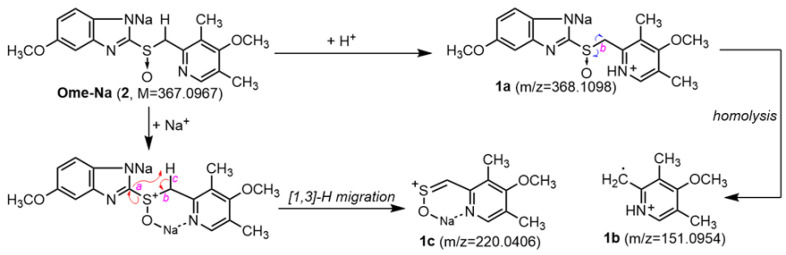
The proposed (+)-ESI–MS fragmentation of Ome-Na (**2**). Full arrows in red color were used to mark the pair electron transformations or fragmentations, while half arrows in blue color were used to mark the single electron movement.

The calculated exact molecular mass of Omeprazole (Ome-H, **1**) was 345.1147, and 367.0967 for its sodium salt (Ome-Na, **2**). The *m*/*z* = 368.1098 was regarded as the [M + H]^+^ (**1a**) of **2** for the positive detecting mode, and it underwent homolytic ionization at position b to give the radical cation **1b** (*m*/*z* = 151.0954) in the HRMS^2^ measurement. [1,3]-H migration in the region a–c of **2** caused the fragmentation and gave a further detectable Na^+^-chelated, 6-membered pyridine fragment **1c** (*m*/*z* = 220.0406). The details are shown in Scheme 1.

#### 2.1.2. 4′-*H*-Ome (**5**) and 4′-*Cl*-Ome (**6**)

**Scheme 2:**
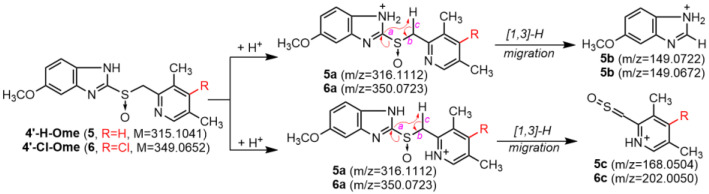
The proposed (+)-ESI-MS fragmentation of 4′-H-Ome (**5**) and 4′-Cl-Ome (**6**). Full arrows in red color were used to mark the pair electron transformations or fragmentations.

The calculated exact molecular mass of 4′-H-Ome (**5**) was 315.1041, and 349.0652 for 4′-*Cl*-Ome (**6**). The *m*/*z* = 316.1112 (**5a**) or 350.0727 (**6a**) was regarded respectively as the [M + H]^+^, which further underwent [1,3]-H migration in the region a–c to give benzoimidazole ion **5b**, and tetravalent sulfur ion **5c** (*m*/*z* = 168.0504) or **6c** (*m*/*z* = 202.0050). The details are shown in Scheme 2.

#### 2.1.3. *N*-Me-Ome (**7**)

**Scheme 3:**
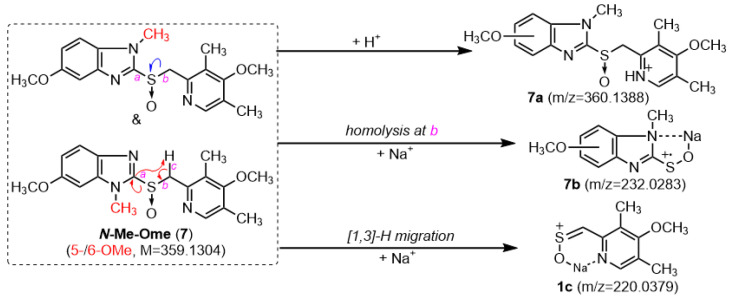
The proposed (+)-ESI-MS fragmentation of *N*-Me-Ome (**7**). Full arrows in red color were used to mark the pair electron transformations or fragmentations, while half arrows in blue color were used to mark the single electron movement.

*N*-Me-Ome (**7**) was a mixture of the two isomers from the isomerized double bond of the imidazole ring. The calculated exact molecular mass of **7** was 359.1304, and the *m*/*z* = 360.1388 was confirmed as [M + H]^+^. The fragmentation pattern was quite different due to the *N*-methylation of the imidazole, and homolysis of **7** occurred at position b to give a detectable Na^+^-chelated, 5-membered benzoimidazole fragment **7b** (*m*/*z* = 232.0283), while [1,3]-H migration in the region a–c gave Na^+^-chelated, 6-membered pyridine ion **1c** (*m*/z = 202.0379). The details are shown in Scheme 3.

#### 2.1.4. *S*-Ome (**8**) and 4′-OH-*S*-Ome (**9**)

**Scheme 4:**
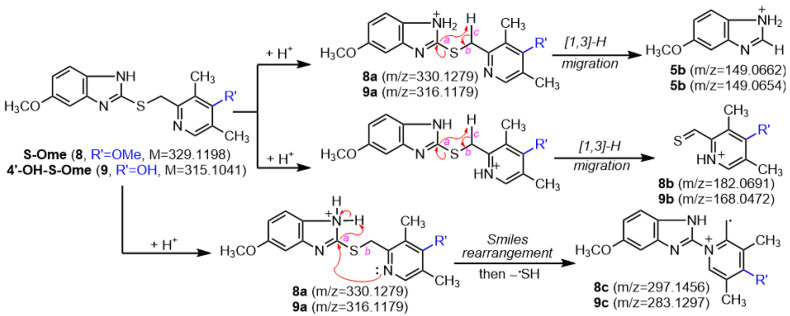
The proposed (+)-ESI-MS fragmentation of S-Ome (**8**) and 4′-OH-S-Ome (**9**). Full arrows in red color were used to mark the pair electron transformations or fragmentations.

The fragmentation ionization of thioethers---S-Ome (**8**) and 4′-OH-S-Ome (**9**) were shown in Scheme 4. The calculated exact molecular mass of **8** was 329.1198, and 315.1041 for **9**. **8a** (*m*/*z* = 330.1279) or **9a** (*m*/*z* = 316.1179) was confirmed as the corresponding [M + H]^+^, which underwent the [1,3]-H migration in the region a–c to give the benzoimidazole cation **5b**, and the pyridine fragment ion **8b** (*m*/*z* = 182.0691) or **9b** (*m*/*z* = 168.0472) containing bivalent sulfur. **8a** or **9a** was fragmented by the attack of the lone pair electrons of the pyridine to start the Smiles rearrangement, followed by a further homolytic cleavage of the C–S bond to give the radical *N*-(2-benzoimdazolyl) pyridine cation **8c** (*m*/*z* = 297.1456), or **9c** (*m*/*z* = 283.1297).

#### 2.1.5. SO_2_-Ome (**11**)

**Scheme 5:**
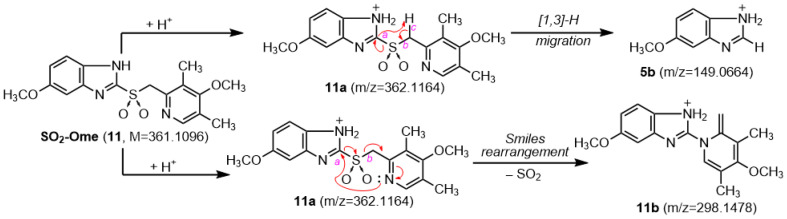
The proposed (+)-ESI-MS fragmentation of SO_2_-Ome (**11**). Full arrows in red color were used to mark the pair electron transformations or fragmentations.

The fragmentation ionization of the sulfone of Omeprazole—SO_2_-Ome (**11**) is depicted in Scheme 5. The calculated exact molecular mass of **11** was 361.1096. **11a** (*m*/*z* = 362.1164) was the [M + H]^+^, which could further fragment via the [1,3]-H migration to the cation **5b**. **11a** was also brought into the Smiles rearrangement and direct extraction from SO_2_ to give the *N*-(2-benzoimdazolyl) pyridine cation **11b** (*m*/*z* = 298.1478). The scaffold of the cation **11b** was very similar to that of **8c** or **9c** while the later was a radical cation.

#### 2.1.6. *N*′-O-Ome (**13**)

**Scheme 6:**
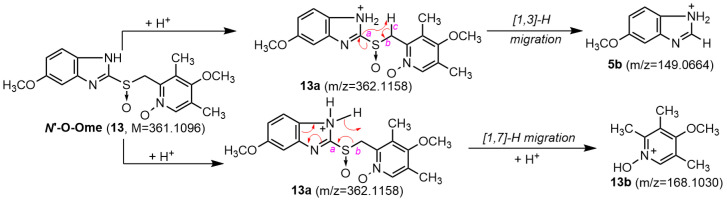
The proposed (+)-ESI-MS fragmentation of *N*′-O-Ome (**13**). Full arrows in red color were used to mark the pair electron transformations or fragmentations.

*N*′-O-Ome (**13**) was an over-oxidized substance related to Omeprazole. The main fragmentation pathway is shown in Scheme 6. The normal fragmentation of its [M + H]^+^ (**13a**) via the [1,3]-H migration to the known cation **5b** was also observed. The rearrangement also occurred across the imidazole ring to give the [1,7]-H migration cation **13b** (*m*/*z* = 168.1030). The [1,7]-H migration was seldom found for these kinds of analogs.

#### 2.1.7. *N*′-O-SO_2_-Ome (**15**)

**Scheme 7:**
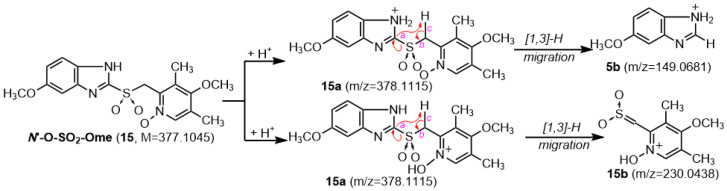
The proposed (+)-ESI-MS fragmentation of *N*′-O-SO_2_-Ome (**15**). Full arrows in red color were used to mark the pair electron transformations or fragmentations.

*N*′-O-SO_2_-Ome (**15**) was another over-oxidized substance related to Omeprazole. The main fragmentation of its [M + H]^+^ ion (**15a**) (Scheme 7) was the [1,3]-H migration to observe the known cation **5b** and the tetravalent sulfur cation **15b** (*m*/*z* = 230.0438). The Smiles rearrangement could not be found due to the absence of the lone pair electrons in the pyridine ring.

### 2.2. Pantoprazole (Panto-H) and the Related Substances

When pantoprazole (Panto-H), pantoprazole sodium (Panto-Na), *N*′-O-Panto (**14**), S-Panto (**10**), SO_2_-Panto (**12**) and *N*′-O-SO_2_-Panto (**16**) were explored by positive ESI-TOF HRMS^1^ and HRMS^2^, similar patterns or results were found, as was the case for Omeprazole and its analogs. These results are summarized in Schemes S1–S5 and Figures S1–S14 in the Supporting Information of this paper.

## 3. Experimental

### 3.1. Apparatus

American Bruker micrOTOF-Q high resolution mass spectrometer.

### 3.2. Drugs and Related Substances

All the compounds were self-prepared in the author’s lab, the synthesis methods were detailed in references [20,21,22] and the structures were confirmed by HRMS, ^1^H NMR and ^13^C NMR. The NMR data were basically consistent with the reported literature [23,24,25].

### 3.3. Spectrometric Condition

The sample was dissolved in methanol and further diluted to around 0.1 μg/mL concentration, and injected into the detector at a flow rate of 0.6 mL/h.

Positive ion electrospray ionization mass spectrum; spray voltage 4500 V; drying gas at a flow rate of 4 L/min with temperature of 180 °C; nebulizer pressure 0.3 bar; mass range 50–1000; cracker voltage 8–22 eV.

## 4. Conclusions

Although the mass spectra of prazoles frequently appeared in the literature, the ionization patterns of the drugs and their related substances were first reported here in a systematic way. Some novel fragmentation routes including H-migration, Smiles rearrangement and metal-chelating ionization were detected, and some interesting ions were newly reported for these kinds of compound in this paper.

### 4.1. Very Common [1,3]-H Migration Ionization

**Figure 2:**
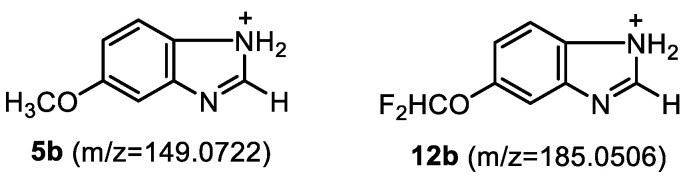
The structures of **5b** and **12b**.

A very common [1,3]-H migration ionization was found for Omeprazole and its analogs to form the benzoimidazole cation **5b** (Figure 2) on the left part of the structures with most of the sulfoxides (**5**, **6** and **13**), thioethers (**8** and **9**), sulfone (**11**), and *N*′-oxide-sulfone (**15**). In the case of pantoprazole and its analogs, a similar ionization was also observed (**12b**, *m*/*z* = 185.0506).

**Figure 3:**
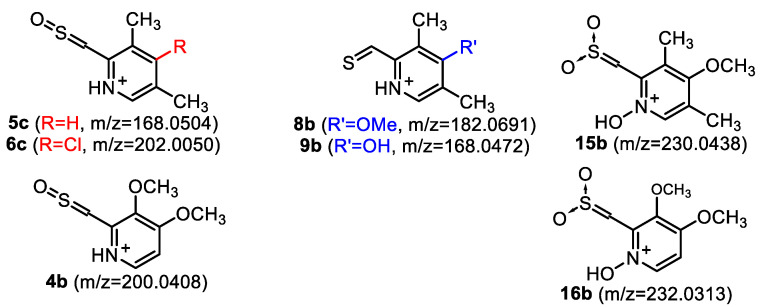
The structures of novel fragment ions were detected as the sulfur cation species.

Interestingly, the right part of the structures of Ar-S(O)_n_CH_2_-Py or *N*′-oxides formed some different kinds of cations via the migration due to the differences in the structures. Some novel and interesting fragment ions were detected as the bivalent (**8b** and **9b,** Figure 3), tetravalent (**5c** and **6c**) or hexavalent (**15b**) sulfur cation species rarely appeared in the literature. Similar phenomena were also found in pantoprazole and its analogs (**4b** and **16b**).

### 4.2. Smiles Rearrangement Ionizations Due to the Attacking of the Lone Pair Electrons

**Scheme 8:**
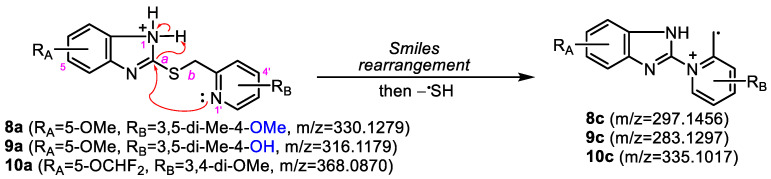
Smiles rearrangement ionizations process of **8a**, **9a**, and **10a**.

Smiles rearrangement ionizations frequently occurred, initiated by the attack of the lone pair electrons from the pyridine ring. For the thioethers (n = m = 0), it could give special *N*-(2-benzoimdazolyl) pyridine radical cations (**8c**, **9c** and **10c,** Scheme 8) via the rearrangement and a further homolytic cleavage of the HS radical.

**Scheme 9:**
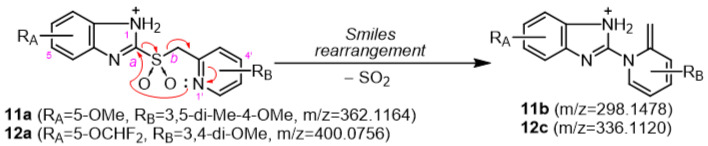
Smiles rearrangement ionizations process of **11a** and **12a**.

And for the sulfones (n = 2, m = 0), it could give similar cations (**11b** and **12c**, Scheme 9) by a direct extraction from SO_2_. However, the sulfoxides were seldom detected in the corresponding Smiles rearrangement ions during our measurement, and the *N*-oxides of the pyridines did not undergo the Smiles rearrangement ionization due to the absence of the lone pair electrons.

### 4.3. [1,7]-H Immigration Could Be Occurred by Crossing the Aromatic Ring

**Scheme 10:**
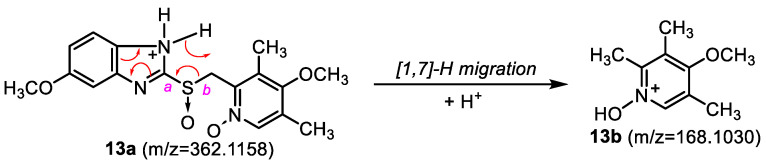
The [1,7]-H migration process of **13a**.

The rearrangement also occurred across the imidazole ring to give the [1,7]-H migration cation **13b** (Scheme 10). This migration was seldom found for these kinds of analogs.

### 4.4. [1,7]-H Immigration Could Be Occurred by Crossing the Aromatic Ring

**Figure 4:**
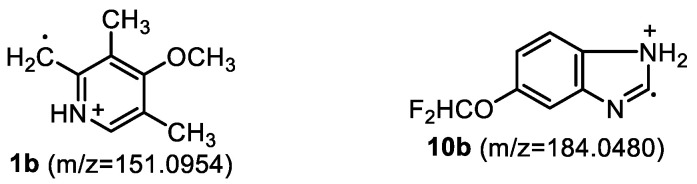
The structures of **1b** and **10b**.

Homolytic fragmentation occasionally occurred on both sides of the functionality -S(O)_n_- of the Ar-S(O)_n_CH_2_-Py. Ome-Na could further fragment to the cation **1b** (Figure 4), while the S-Panto could give the cation **10b**.

### 4.5. 5/6-Membered Chelating Cations with Na^+^ or K^+^

**Figure 5:**
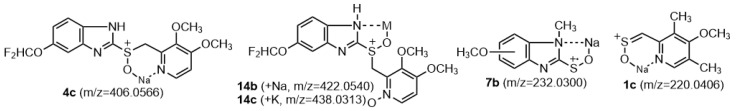
The structures of the 5/6-membered chelating cations of the molecule with Na^+^ or K^+^, or further fragment cations.

The 5/6-membered chelating cations of the molecule with Na^+^ or K^+^ (Figure 5), or further fragment cations, were frequently found for our study. The fragmentation ion of the left part appeared as a 5-membered chelating ion (**7b**, **14b** and **14c**), and the right pyridine ring was found as the 6-membered chelating ion (**1c** and **4c**).

## Data Availability

Data are available within the article and Supplementary Materials.

## Supplementary Materials

The following supporting information can be downloaded at: https://www.mdpi.com/article/10.3390/molecules28155852/s1, Schemes S1–S5 The tested results and analysis of the related substances; Figures S1–S14: The HRMS^1^ and HRMS^2^ of the related substances.
