# Supplementary material for: A Mechanism Study on the (+)-ESI-TOF/HRMS Fragmentation of Some PPI Prazoles and Their Related Substances
Source: Molecules. 2023 Aug 3;28(15):5852. doi: 10.3390/molecules28155852 (PMC10420917; doi:10.3390/molecules28155852)
Supplement: Supplementary file 1 [file molecules-28-05852-s001.zip › molecules-2417573-supplementary.pdf]

The mechanism study on the (+)–ESI–TOF/HRMS fragmentation of some PPI Prazoles and their related substances

Luhong Wang, Lixue Chen, Yichen Yao, Hongyan Shen* and Youjun Xu*

School of Pharmaceutical Engineering, and Key Laboratory of Structure-Based Drug Design & Discovery (Ministry of Education), Shenyang Pharmaceutical University, Shenyang 110016, PR China.

**Corresponding author.*

Email address: hyshen@syphu.edu.cn (H. Shen);

Email address: xuyoujun@syphu.edu.cn (Y. Xu).

Supporting information

1. Omeprazole (Ome-Na) and the related substances

1.1 Omeprazole (Ome-Na): The mass spectrum in positive ion mode was shown in **Figure S1**.

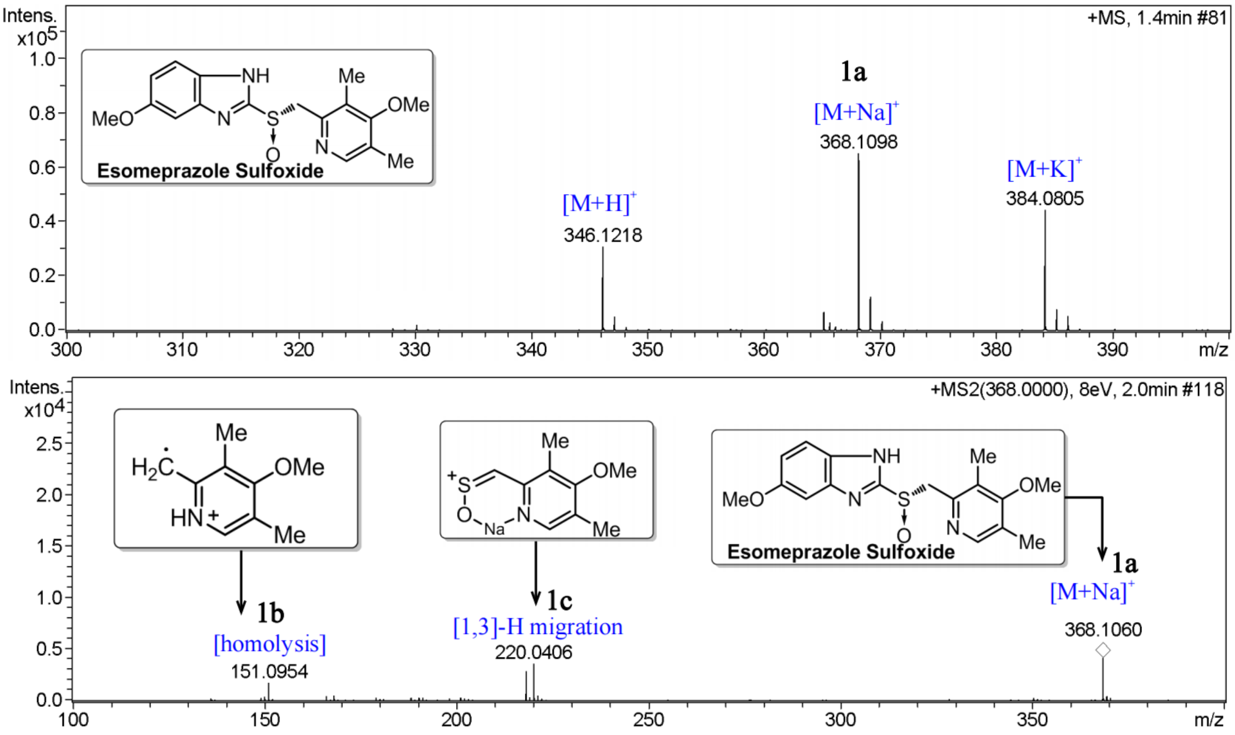

Figure S1. The HRMS[1] and HRMS[2] of Ome-Na.

*1.2 4'-H-Ome (**5**) and 4'-Cl-Ome (**6**)*: The mass spectrum in positive ion mode was shown in **Figure S2** and **Figure S3**.

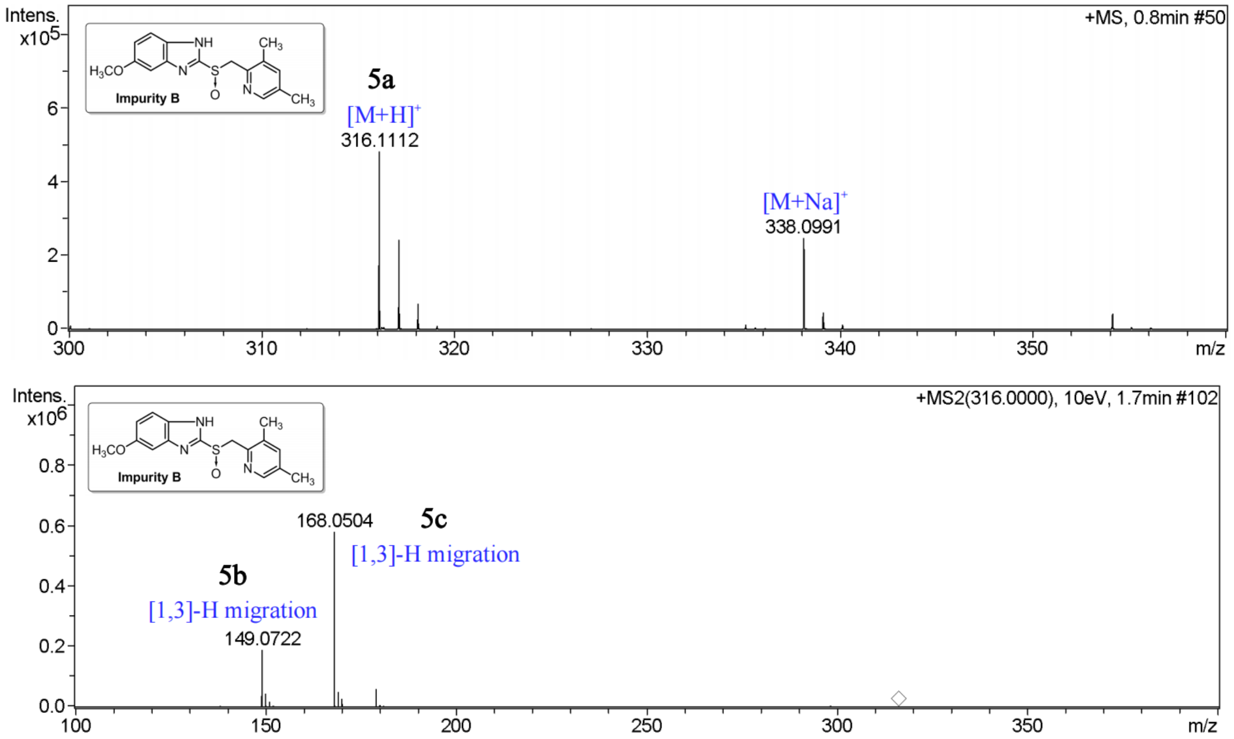

Figure S2. The HRMS[1] and HRMS[2] of **5**.

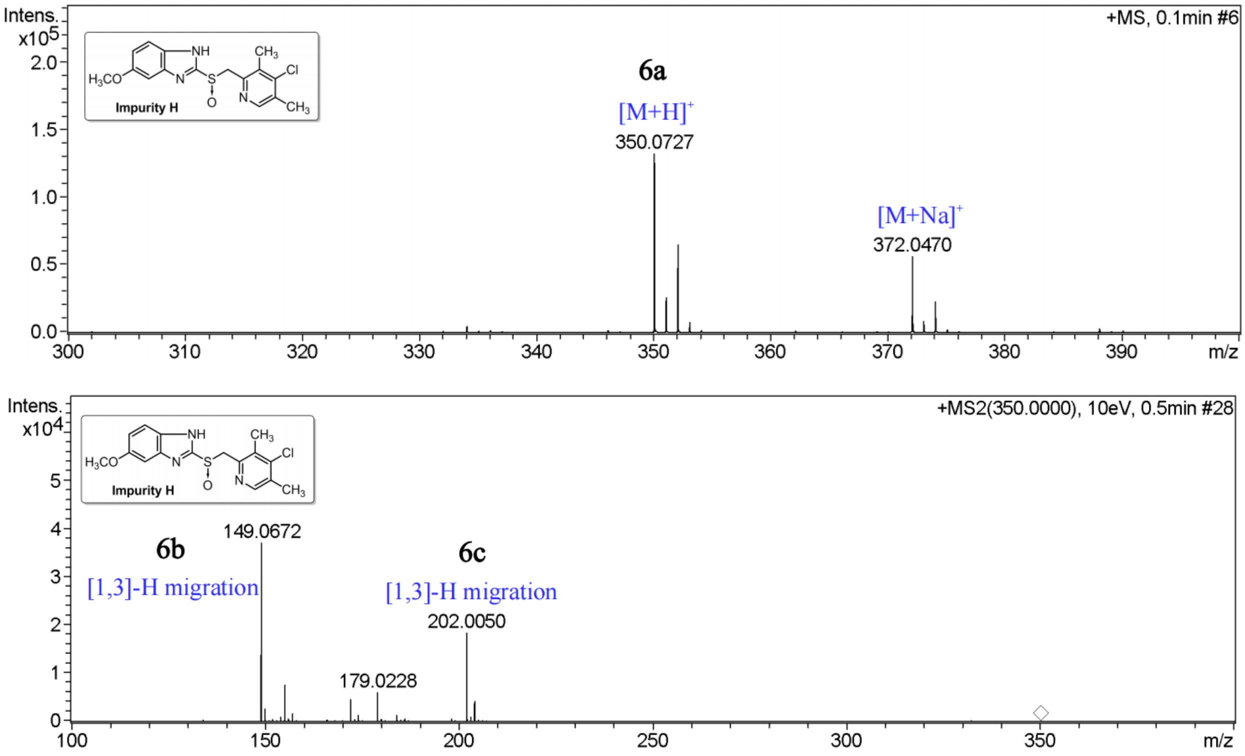

Figure S3. The HRMS[1] and HRMS[2] of **6**.

1.3 N-Me-Ome (7): The mass spectrum in positive ion mode was shown in **Figure S4**.

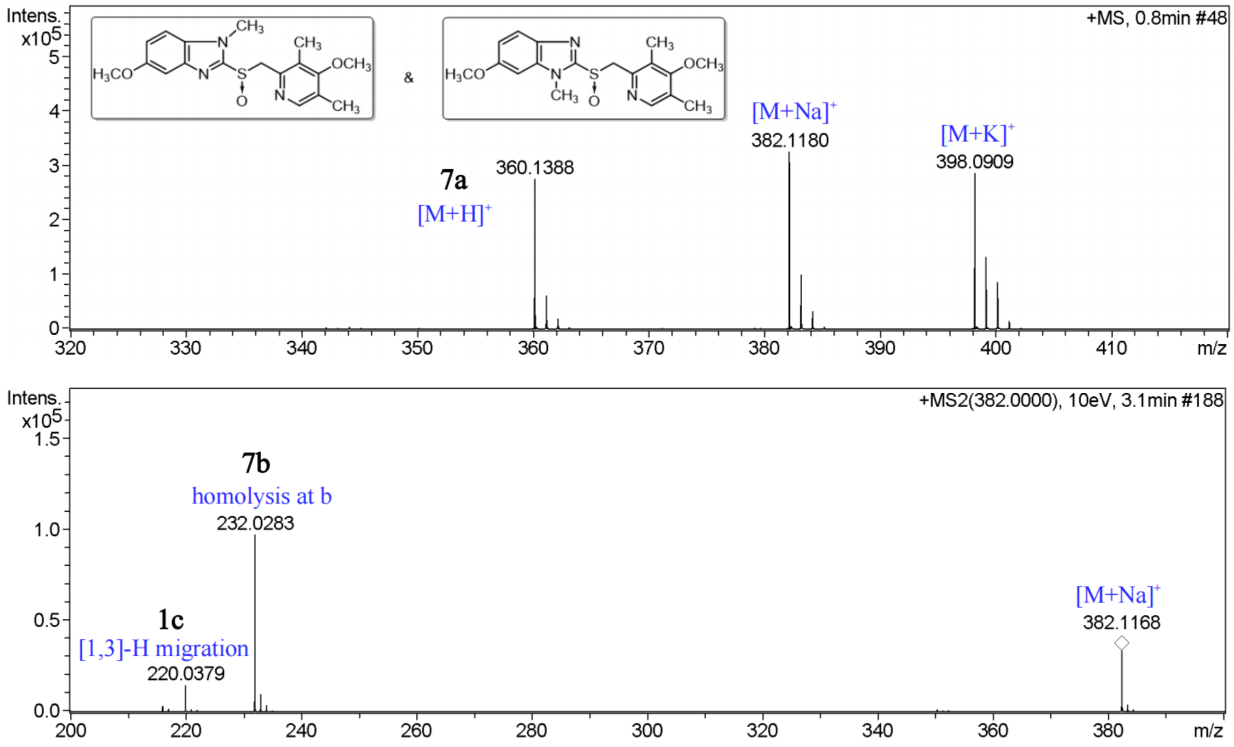

Figure S4. The HRMS[1] and HRMS[2] of **7**.

*1.4 S-Ome (**8**) and 4'-OH-S-Ome (**9**)*: The mass spectrum in positive ion mode was shown in **Figure S5** and **Figure S6**.

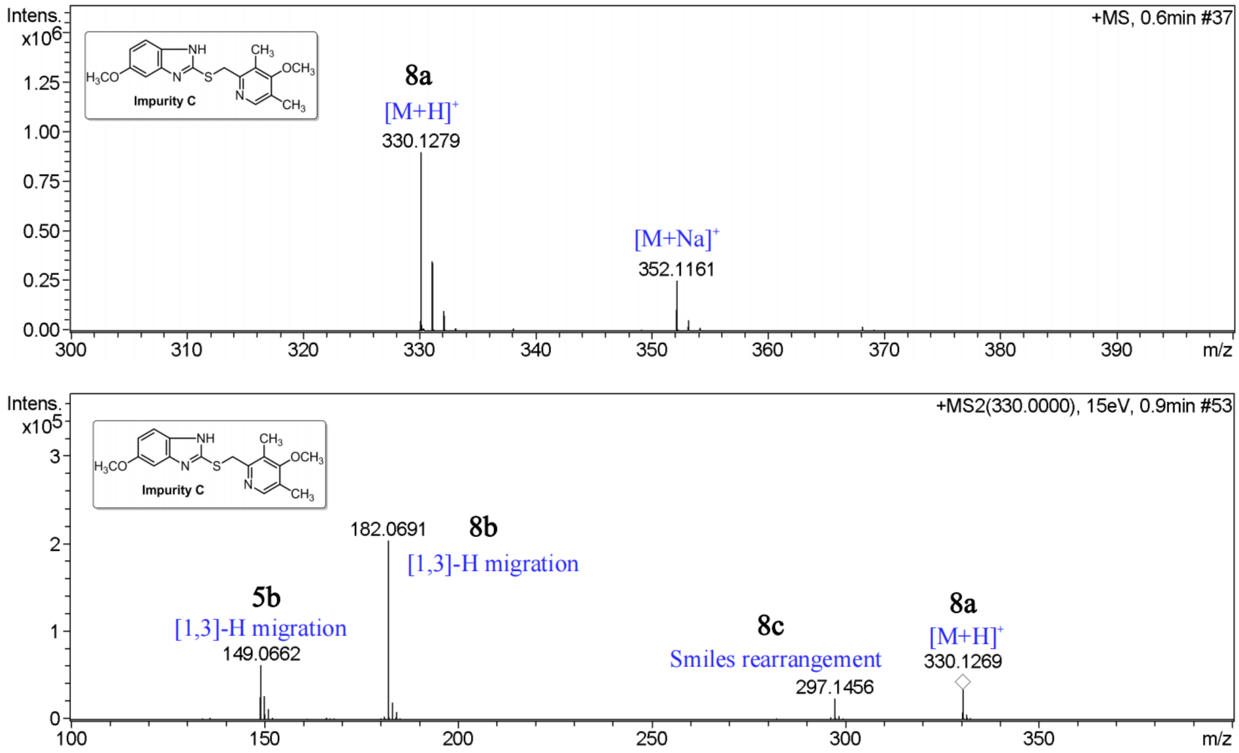

Figure S5. The HRMS[1] and HRMS[2] of **8**.

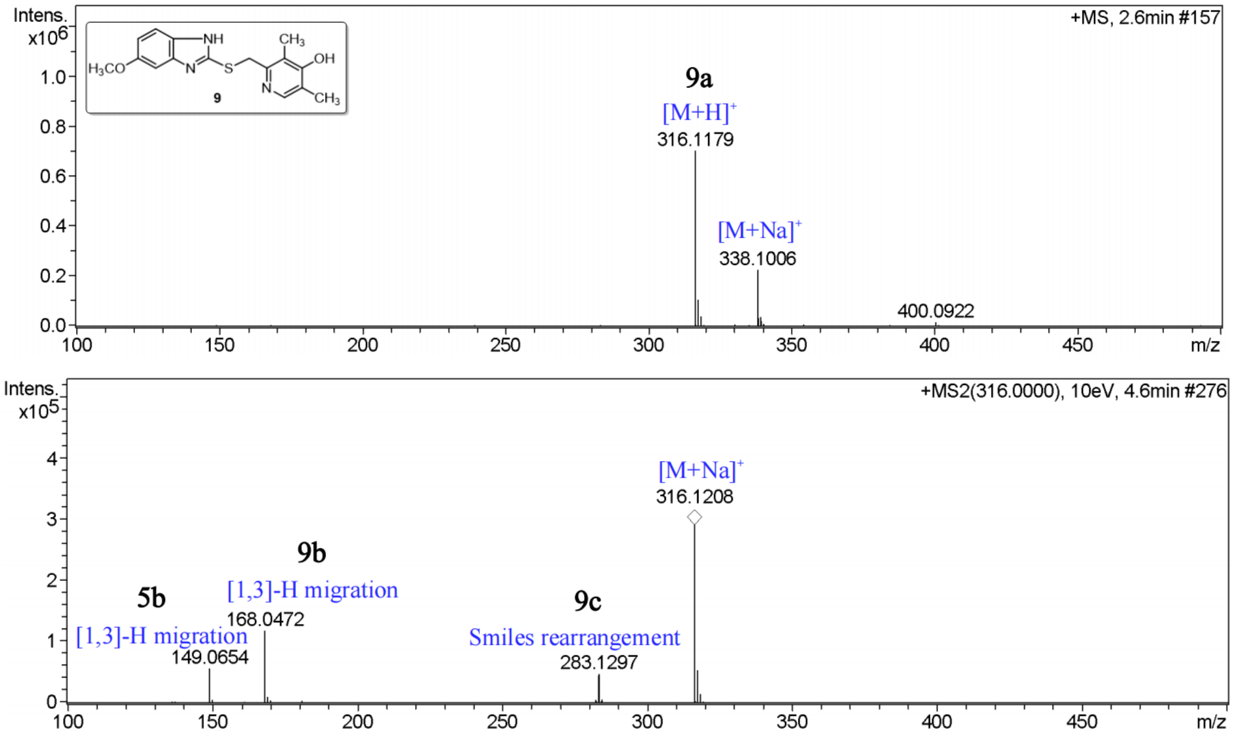

Figure S6. The HRMS[1] and HRMS[2] of **9**.

*1.5 SO_2-Ome (**11**)*: The mass spectrum in positive ion mode was shown in **Figure S7**.

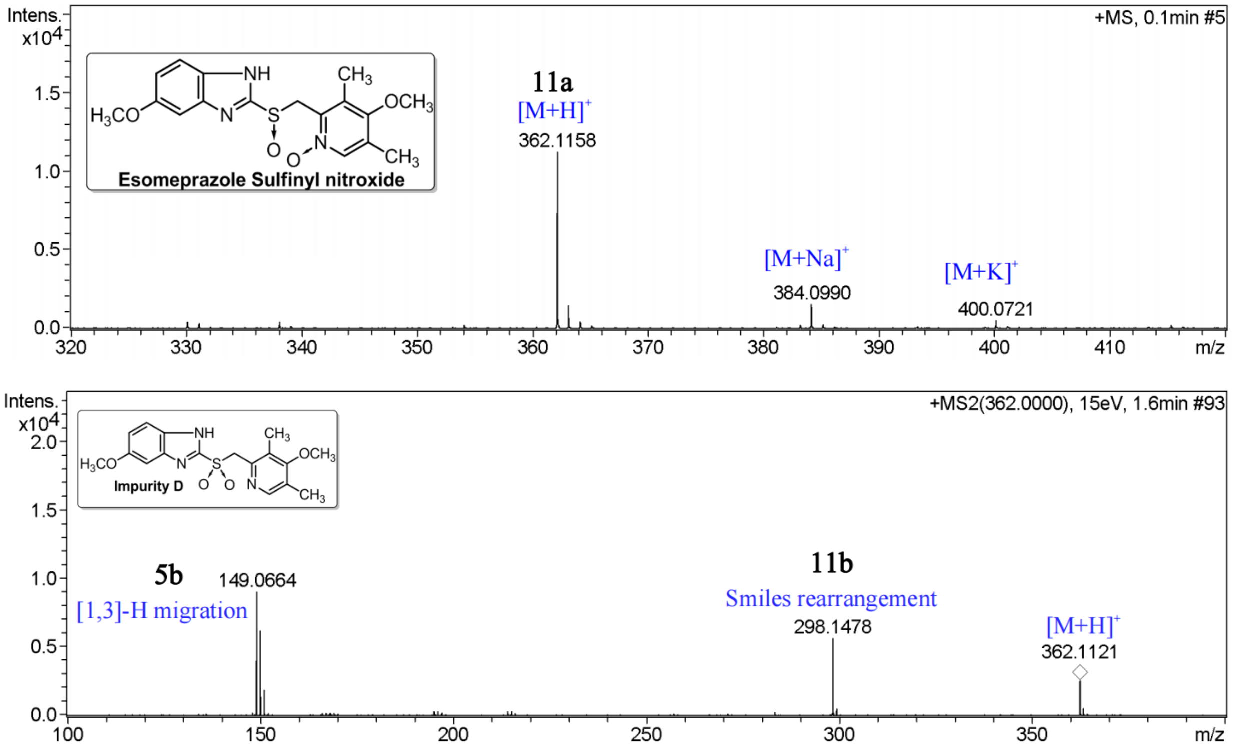

Figure S7. The HRMS[1] and HRMS[2] of **11**.

*1.6 N'-O-Ome (**13**)*: The mass spectrum in positive ion mode was shown in **Figure S8**.

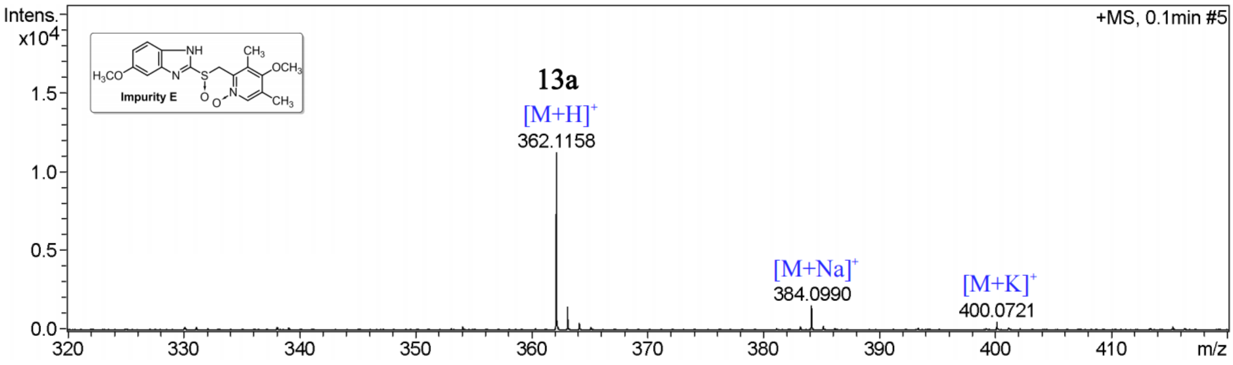

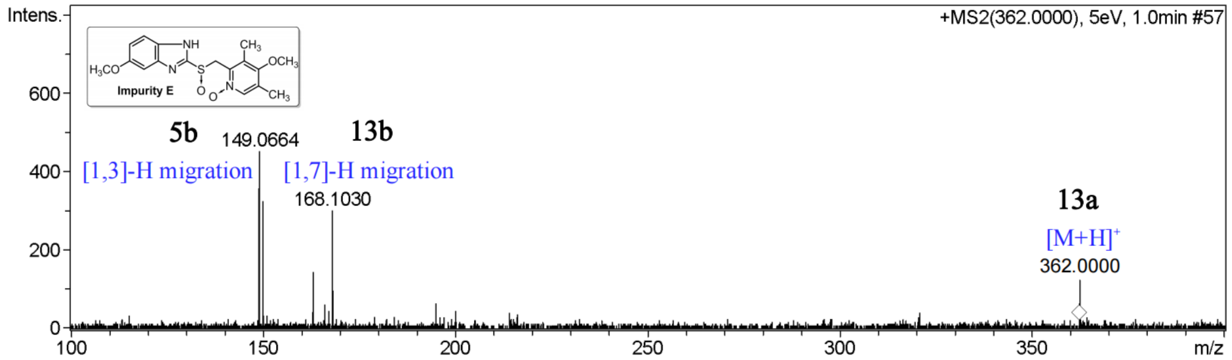

Figure S8. The HRMS[1] and HRMS[2] of **13**.

*1.7 N'-O-SO$_2$-Ome (**15**)*: The mass spectrum in positive ion mode was shown in **Figure S9**.

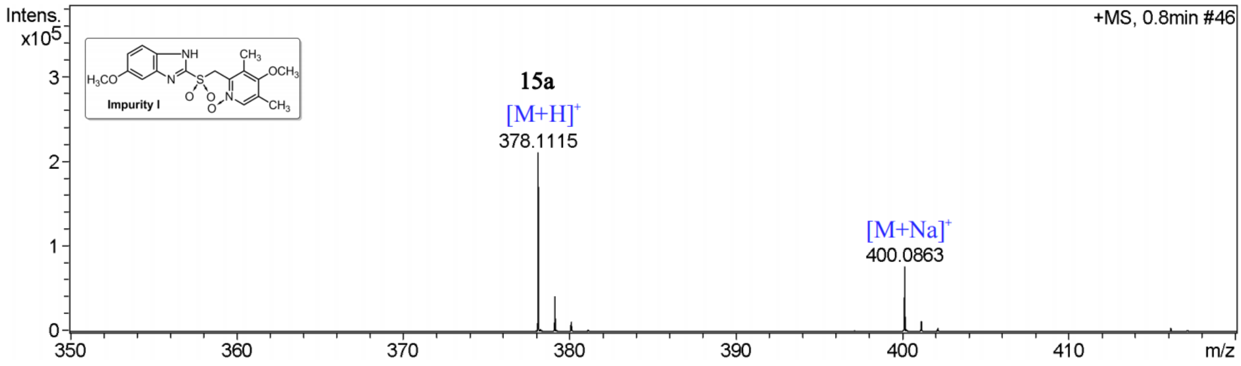

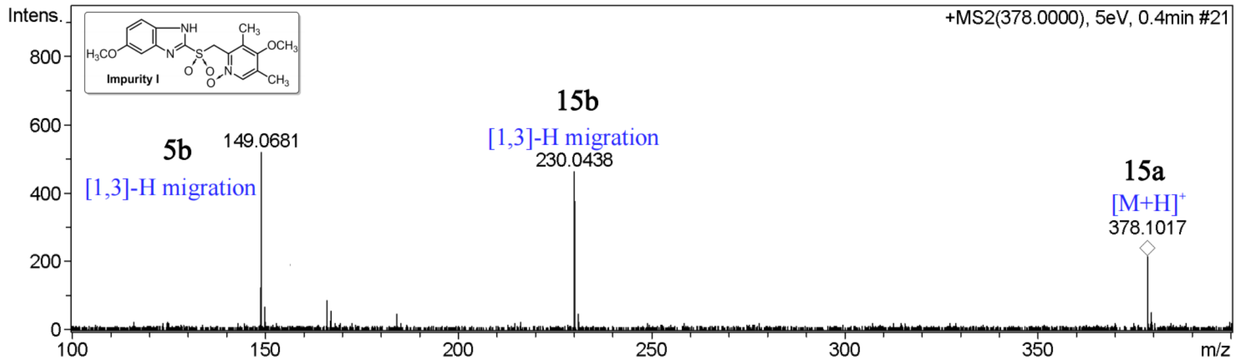

Figure S9. The HRMS[1] and HRMS[2] of **15**.

2. Pantoprazole (Panto-H) and the related substances

2.1 Pantoprazole (Panto-H) and Pantoprazole Sodium (Panto-Na): The mass spectrum in positive ion mode was shown in **Figure S10**, and the tested results and analysis were displayed in **Scheme S1**.

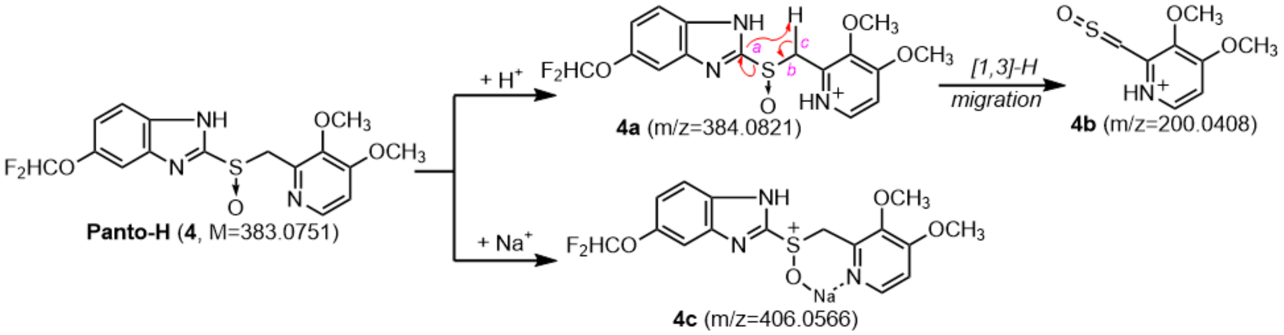

Scheme S1. The proposed (+)-ESI-MS fragmentation of Panto-H (**4**).

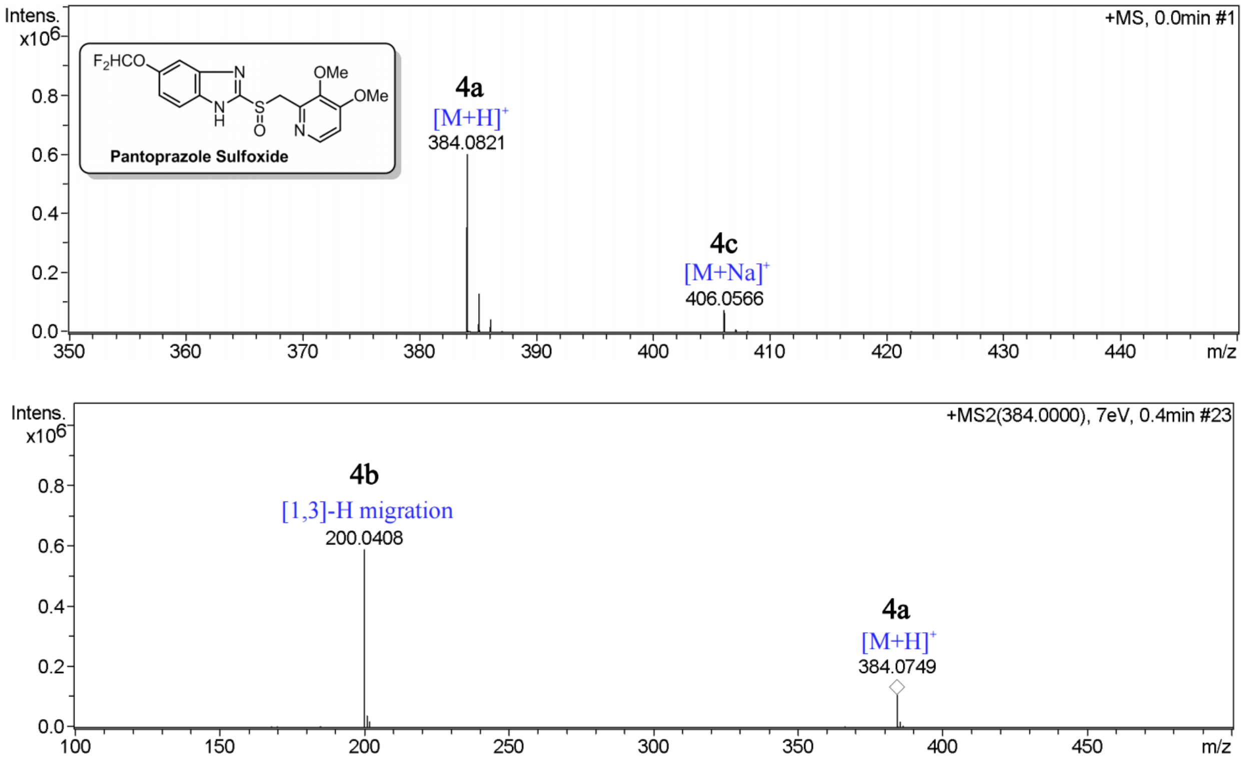

Figure S10. The HRMS[1] and HRMS[2] of Panto-H (**4**).

2.2 N'-O-Panto (14): The mass spectrum in positive ion mode was shown in **Figure S11**, and the tested results and analysis were displayed in **Scheme S2**.

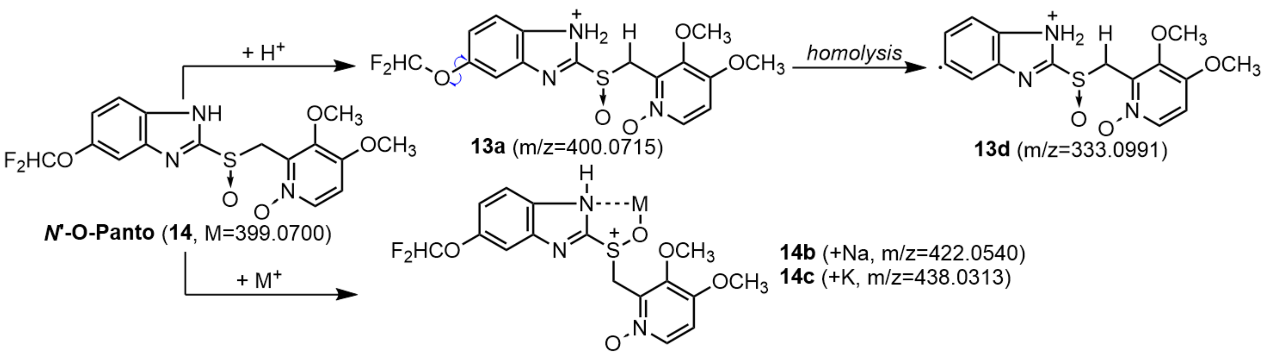

Scheme S2. The proposed (+)-ESI-MS fragmentation of *N'*-O-Panto (**14**).

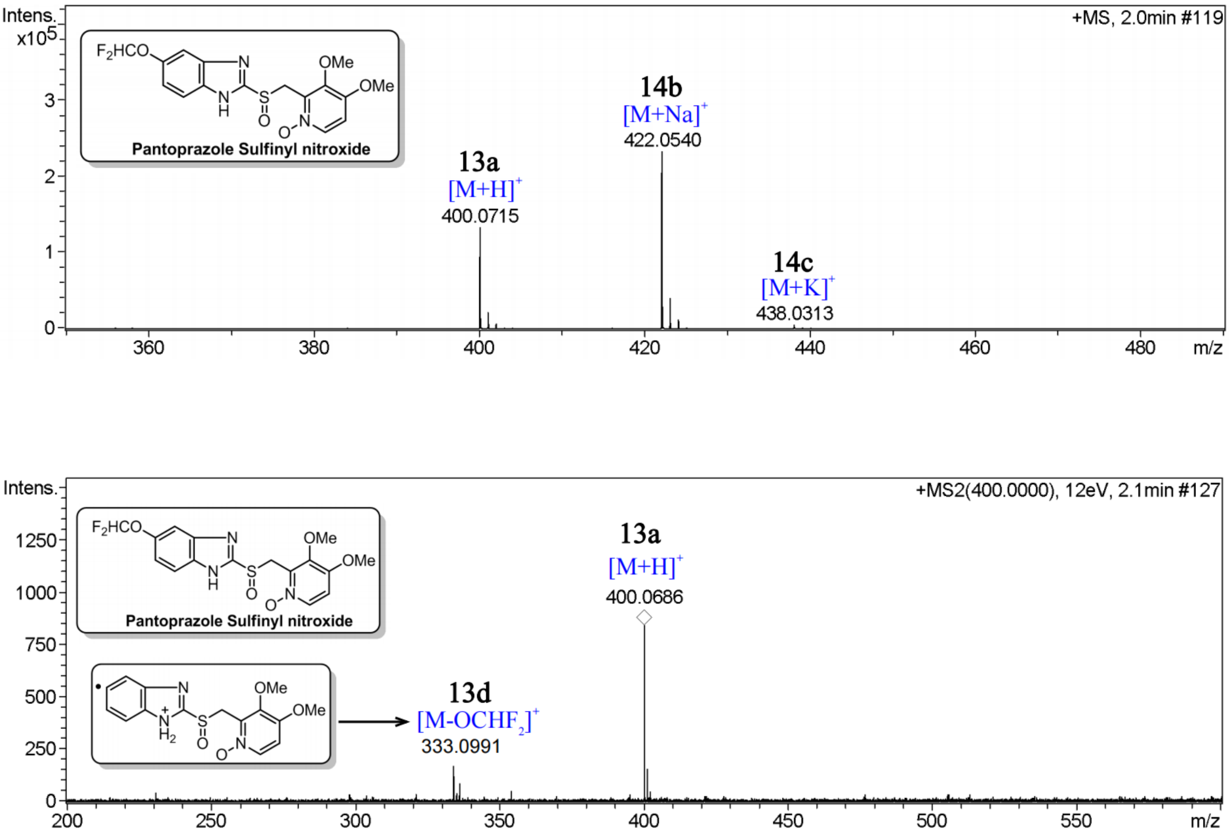

Figure S11. The HRMS[1] and HRMS[2] of **14**.

2.3 S-Panto (10): The mass spectrum in positive ion mode was shown in **Figure S12**, and the tested results and analysis were displayed in **Scheme S3**.

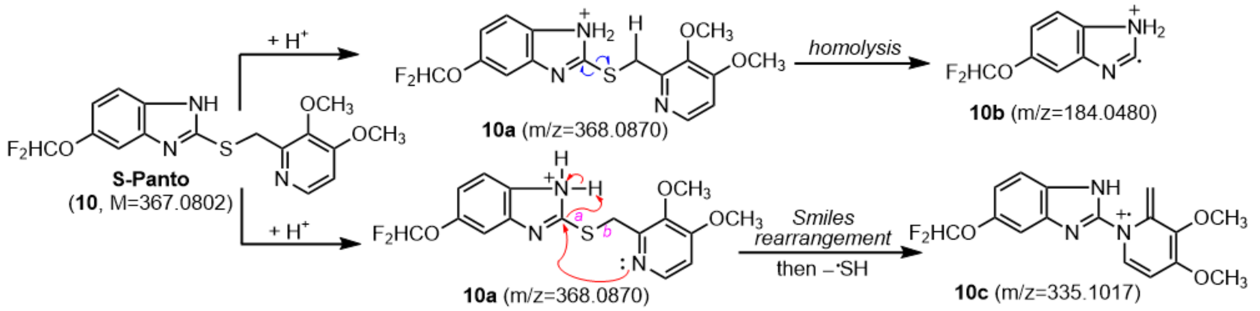

Scheme S3. The proposed (+)-ESI-MS fragmentation of S-Panto (**10**).

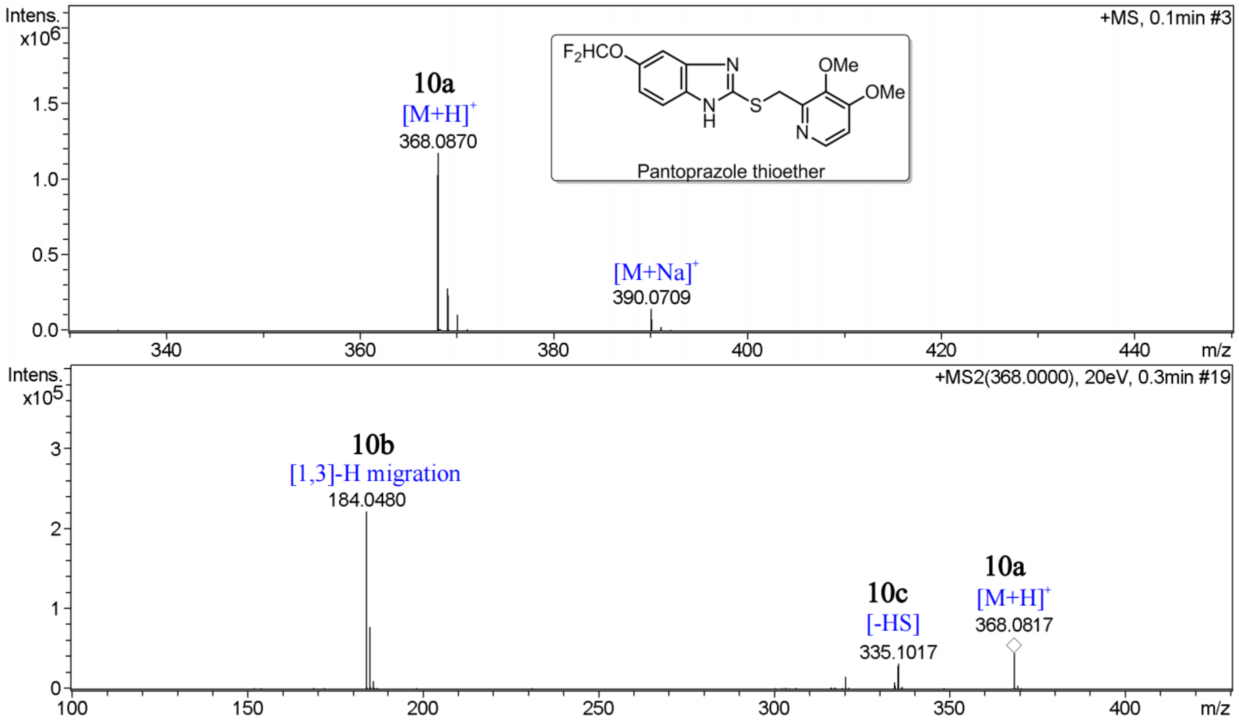

Figure S12. The HRMS[1] and HRMS[2] of **10**.

*2.4 SO_2-Panto (**12**)*: The mass spectrum in positive ion mode was shown in **Figure S13**, and the tested results and analysis were displayed in **Scheme S4**.

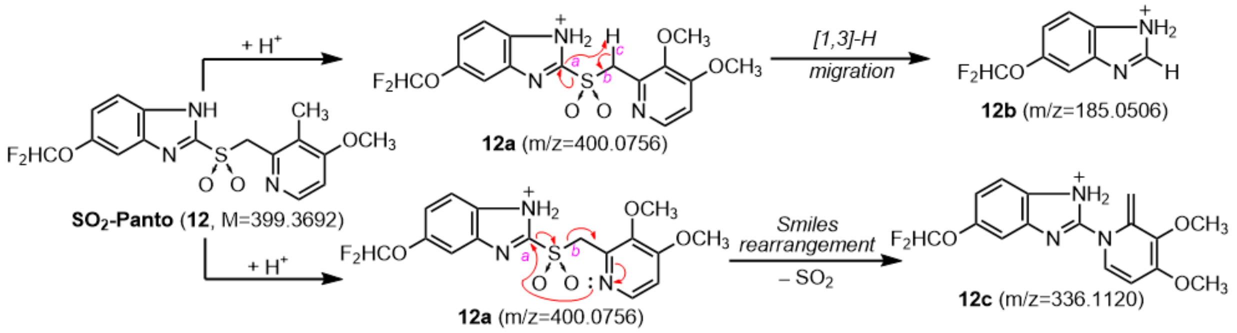

Scheme S4. The proposed (+)-ESI-MS fragmentation of SO_2-Panto (**12**).

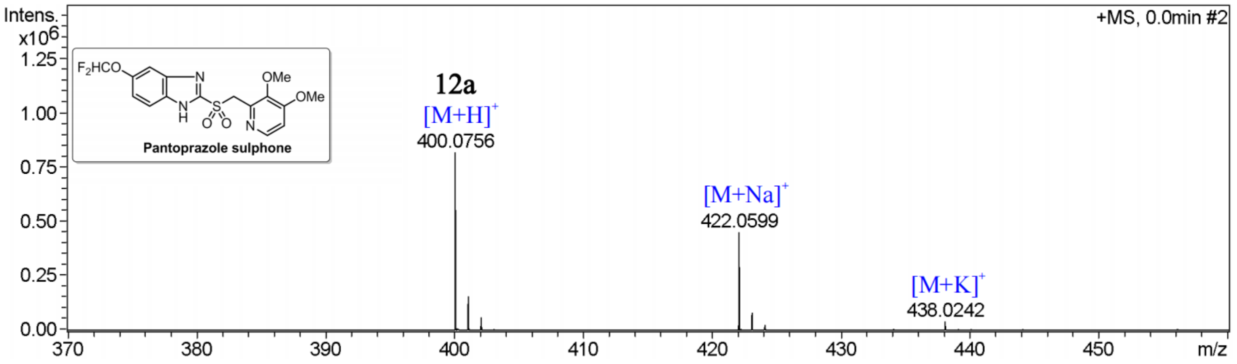

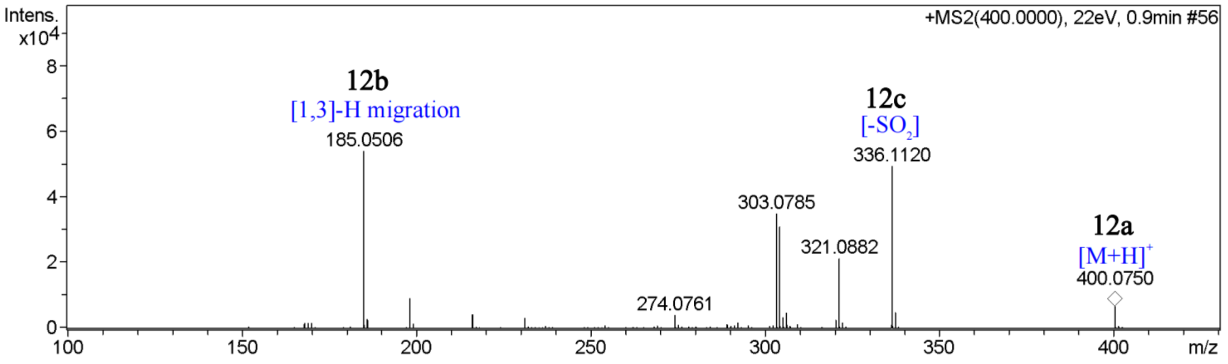

Figure S13. The HRMS[1] and HRMS[2] of **12**.

*2.5 N'-O-SO₂-Panto (**16**)*: The mass spectrum in positive ion mode was shown in **Figure S14**, and the tested results and analysis were displayed in **Scheme S5**.

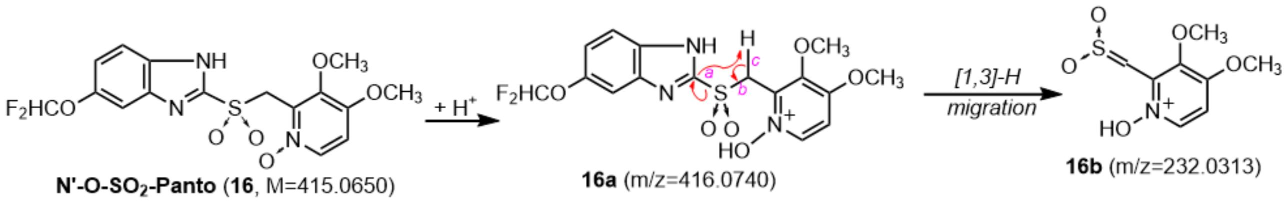

Scheme S5. The proposed (+)-ESI-MS fragmentation of N'-O-SO_2-Panto (**16**).

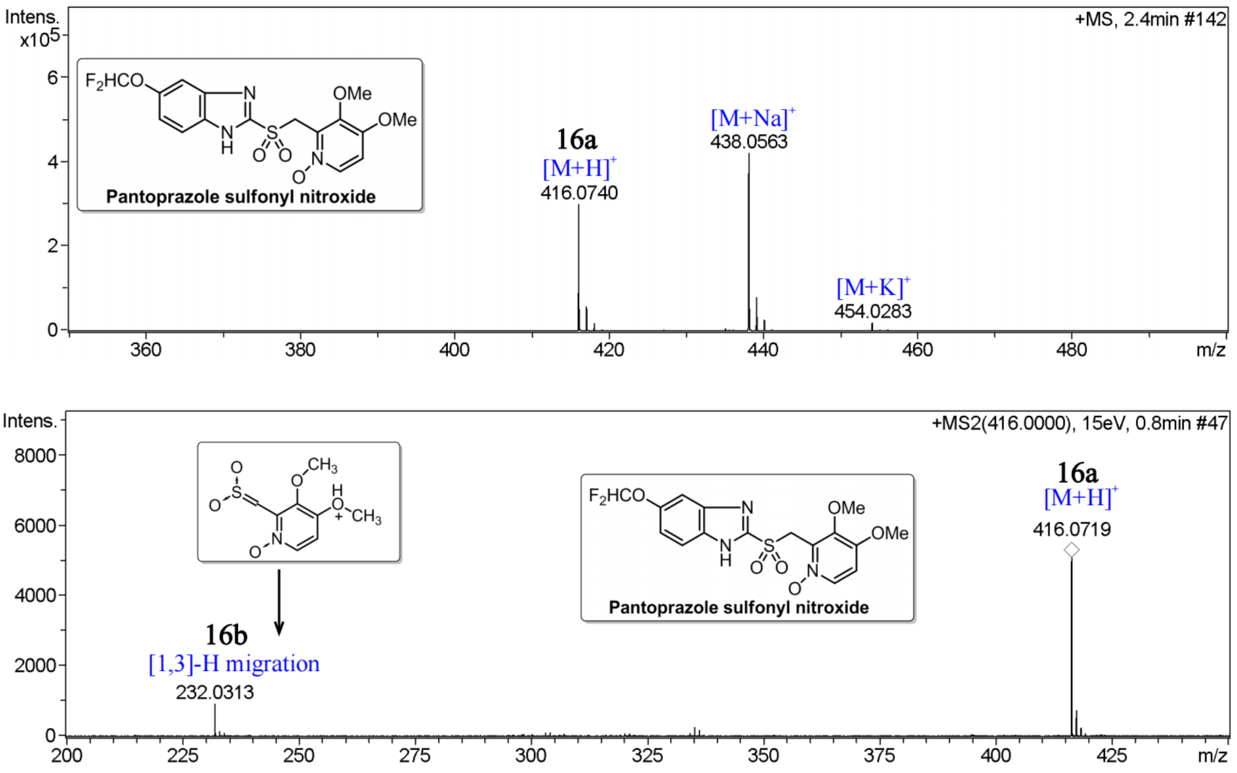

Figure S14. The $HRMS^1$ and $HRMS^2$ of **16**.
